# Long-term prognosis of unrecognized myocardial infarction detected with cardiovascular magnetic resonance in an elderly population

**DOI:** 10.1186/s12968-016-0264-z

**Published:** 2016-07-19

**Authors:** Charlotte Ebeling Barbier, Raquel Themudo, Tomas Bjerner, Lars Johansson, Lars Lind, Håkan Ahlström

**Affiliations:** Department of Radiology, Uppsala University Hospital, Uppsala, 751 85 Sweden; Department of Medicine, Uppsala University Hospital, Uppsala, Sweden

**Keywords:** Unrecognized myocardial infarction, Cardiovascular magnetic resonance, Epidemiology, Prognosis

## Abstract

**Background:**

Individuals with unrecognized myocardial infarctions (UMIs) detected with cardiovascular magnetic resonance (CMR) constitute a recently defined group whose prognosis has not been fully evaluated. However, increasing evidence indicate that these individuals may be at considerable cardiovascular risk. The aim of the present study was to investigate the prognostic impact of CMR detected UMIs for major adverse cardiac events (MACE) in community living elderly individuals.

**Methods:**

Late gadolinium enhancement CMR was performed in 248 randomly chosen 70-year-olds. Individuals with myocardial infarction (MI) scars, with or without a hospital diagnosis of MI were classified as recognized MI (RMI) or UMI, respectively. Medical records and death certificates were scrutinized. MACE was defined as cardiac death, non-fatal MI, a new diagnosis of angina pectoris, or symptom-driven coronary artery revascularization.

**Results:**

During follow-up (mean 11 years) MACE occurred in 10 % (*n* = 18/182) of the individuals without MI scars, in 20 % (*n* = 11/55) of the individuals with UMI, and in 45 % (*n* = 5/11) of the individuals with RMI, with a significant difference between the UMI group and the group without MI scars (*p* = 0.045), and between the RMI group and the group without MI scars (*p* = 0.0004). Cardiac death and/or non-fatal MI occurred in 15, 5, and 3 of the individuals in the NoMI, UMI, and RMI group respectively. Hazards ratios for MACE adjusted for risk factors and sex were 2.55 (95 % CI 1.20-5.42; *p* = 0.015) for UMI and 3.28 (95 % CI1.16-9.22; *p* = 0.025) for RMI.

**Conclusions:**

The presence of a CMR detected UMI entailed a more than double risk for MACE in community living 70-year-old individuals.

## Background

During a century the prevalence of unrecognized myocardial infarctions (UMIs) has been estimated with electrocardiography (ECG), using a persistent Q-wave as sign of MI [1]. However, late gadolinium enhancement cardiovascular magnetic resonance (LGE-CMR) detects more UMIs than ECG [2], by depicting non-viable myocardium [3]. Thus, ECG-detected and CMR detected UMIs are not the same UMIs [2, 4, 5]. The prognosis of an ECG-detected UMI is similar to that of a recognized myocardial infarction (RMI) [6–8], whereas individuals with CMR detected UMIs constitute a recently defined group whose prognosis has not been as thoroughly investigated. However, increasing evidence indicate that these individuals may be at considerable cardiovascular risk [5, 9–14]..

A number of studies have been conducted in selected risk populations establishing CMR detected UMIs as an important risk factor for major adverse cardiac events (MACE). This has been demonstrated in patients with confirmed [9, 10] or suspected [9–11] coronary artery disease, in patients with peripheral arterial occlusive disease [13], and in diabetic [12] and prediabetic [14] patients.

Only one epidemiology study on the prognostic impact of CMR detected UMIs has been published. It was performed in a mixed cohort with randomly chosen participants and diabetes patients and it reveals an increased all-cause mortality risk in elderly individuals with UMI compared to those without MI [5]. To the best of our knowledge, this is the first study to investigate the relationship between CMR detected UMIs and cardiac death or other severe cardiac events in long-term follow-up of an entirely population based cohort.

The aim of the present study was to investigate the prognostic impact of CMR detected UMIs on cardiac events in community living elderly individuals.

## Methods

### Study population

CMR was performed on an unselected subsample from the Prospective Investigation of the Vasculature in Uppsala Seniors (PIVUS) study [15]. Eligible for the PIVUS study were all individuals aged 70 years and resident in the municipality of Uppsala, Sweden. The individuals were chosen in a randomized manner from the register of municipality inhabitants, and 2025 individuals were invited to participate within weeks from their 70th birthday; 1016 agreed and gave written informed consent.

From the original cohort, 283 individuals were consecutively invited to undergo CMR, which was finally performed on 259 individuals [2]. Their mean age was 71 years and 6 months (range 70 years, 5 months to 71 years, 10 months) when CMR was performed during the years 2003–2005. The number of invited individuals was preset, determined by financial limitations and the availability of CMR scan time. Eleven examinations were excluded because of poor image quality, leaving assessable data from 248 individuals (123 women, 125 men).184 of these individuals were reexamined five years later (17 had died, 47 declined to participate).

The basic characteristics and major cardiovascular risk factors of these individuals have been described elsewhere [2] and did not differ significantly from those in the entire PIVUS population [15], except that there were fewer current smokers among the individuals of the present study. The cardiac morbidity of the PIVUS study participants did not differ significantly from that of the background population [15].

### Participant data and definitions

Medical records from all divisions of Uppsala University Hospital and from all general practitioners in the county were scrutinized by a doctor in September 2015 and data on cardiac and atherosclerotic symptoms, morbidity and mortality that occurred after the CMR examination were collected. In cases when the individual was registered to be deceased in the medical records, death certificates were obtained and reviewed.

MACE was defined as cardiac death (i.e. cardiac arrest being registered as the primary cause of death in the death certificate), non-fatal MI (i.e. a hospital diagnosis of MI set using the criteria defined by the Joint European Society of Cardiology/American College of Cardiology Committee [16]), a new diagnosis of angina pectoris, or symptom-driven coronary artery revascularization. Only diagnoses and events acquired or occurring after CMR were considered. In individuals with several MACEs, the time between CMR and the first MACE was documented. The term other cardiac morbidity includes diagnoses of arrhythmias, congestive heart failure, or valvular disorders. The term other atherosclerotic disease includes diagnoses of carotid stenosis, renal artery stenosis, or peripheral arterial disease.

### Image acquisition and analysis

MR image acquisition and analysis has been described elsewhere [2]. Briefly, a 1.5 Tesla MR system was used (Gyroscan Intera, Philips Medical Systems, Best, the Netherlands) to acquire late gadolinium enhancement images after injection of 40 ml Gd-DTPA-BMA (OmniscanTM, GE Healthcare, Oslo, Norway). At 70 years of age 40 ml gadolinium-diethylenetriamine pentaacetic acid-bismethylamide (OmniscanTM, GE Healthcare, Oslo, Norway) was administered in all individuals since a whole body MR angiography was performed prior to acquiring the late gadolinium enhancement images. At 75 years of age the contrast dose was adjusted to body weight (0.2 mmol/kg).

Late gadolinium enhancement (LGE) images were acquired using a 3D inversion recovery gradient echo sequence covering the entire heart in short and long axis views. The acquired slice thickness was 10 mm with a resolution of 1.56 x 2.81 mm and the inversion time was individually adjusted. Cine images were acquired during breath holding using asteady state free precession sequence as previously described [2, 17].

LGE images were assessed by two radiologists independently and in a consensus reading, using subendocardial involvement as a criterion for identifying MI scars [18, 19]. The observers were blinded to each other’s assessments and to information on any previous disease as described elsewhere [2, 20]. The radiologists who analyzed the images acquired when the individuals were 75 years old were blinded to the analysis results from images acquired when the individuals were 70 years old. To avoid over reporting the prevalence of MI scars, an additional consensus reading was performed in which images displaying MI scars at either 70 or 75 years of age were compared side by side with the images from the individual’s other MR examination and viability was assessed also taking information from the cine images into consideration.

The individuals were grouped based on the LGE-CMR findings on images acquired when the individuals were 70 years old and the data from medical records: 182 had no MI scar, 55 had a UMI (i.e. an MI scar [18, 19] but no MI diagnosis), and 11 had an RMI (i.e. an MI scar in combination with an MI diagnosis in medical records).

### Statistical analysis

StatView version 5.0.1 (SAS Institute, Cary, North Carolina) was used for statistical analyses. The Chi^2^-test was used for estimating differences between groups. Cox’s Proportional Hazards Model and the Kaplan-Meier method were used to estimate event-free survival in the three groups. Improvement in discrimination by adding information on UMI to the Framingham risk score was calculated with C-statistics. The significance level was set at 0.05 in the primary analysis, i.e. the comparison between the UMI and the NoMI groups. The comparison between the RMI and the NoMI groups was regarded as a secondary analysis.

### Results

The mean follow-up time was 11 years (range 9 years and 10 months – 12 years and 7 months). No individuals were lost to follow-up. Forty-eight of the 248 individuals were deceased, 6 from cardiac arrest and 42 from non- cardiac reasons (i.e. 17 from cancer, 4 from stroke, 6 from dementia or other degenerative disease, 5 from trauma, and10 from infectious disease and/or organ failure).

**Fig. 1:**
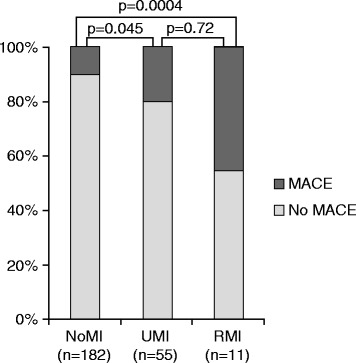
Distribution of MACE. The distribution of major adverse cardiac events (MACE) (dark grey) in individuals without MI scars (NoMI) on CMR in individuals with CMR detected unrecognized myocardial infarctions (UMI), and in individuals with recognized myocardial infarction (RMI). *p*-values of the differences are displayed in the figure

During follow-up, MACE occurred in 10 % (*n* = 18/182) of the individuals without MI scars, in 20 % (*n* = 11/55) of the individuals with UMI, and in 45 % (*n* = 5/11) of the individuals with RMI, with significant differences between the UMI group and the group without MI scars (*p* = 0.045) and between the RMI group and the group without MI scars (*p* = 0.0004). (Fig. 1) In sex-specific analyses this difference was only significant between the RMI group and the group without MI scars in men (*p* = 0.0013). The distribution of MACE between the groups and sexes is displayed in Table 1. Cardiac death and/or non-fatal MI occurred in 15, 5, and 3 of the individuals in the NoMI, UMI, and RMI group respectively.

**Table 1:** Distribution of major adverse cardiac events (MACE) between the sexes in individuals with or without myocardial scars on cardiac magnetic resonance imaging

	No MI (*n* = 182)		UMI (*n* = 55)		RMI (*n* = 11)	
	Men (*n* = 84)	Women (*n* = 98)	Men (*n* = 32)	Women (*n* = 23)	Men (*n* = 9)	Women (*n* = 2)
MACE, number (%)	11 (13)	7 (7)	9 (28)	2 (9)	5 (6)	0

*No MI* no MI scar, *RMI* recognized myocardial infarction, i.e., MI scar in combination with MI diagnosis in medical records, *UMI* unrecognized myocardial infarction

**Fig. 2:**
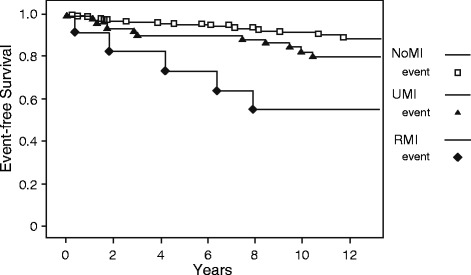
Event-free survival. Kaplan-Meier estimation of event-free survival in individuals without MI scars (NoMI) on cardiac magnetic resonance imaging, in individuals with CMR detected unrecognized myocardial infarctions (UMI), and in individuals with recognized myocardial infarction (RMI)

The unadjusted hazards ratio (HR) for MACE for individuals with UMI was 2.62; 95 % confidence interval (CI) 1.24-5.55; *p* = 0.012. The unadjusted HR for individuals with RMI was 6.14; 95 % CI 2.27-16.6; *p* = 0.0003. A Kaplan-Meier estimation of event-free survival is displayed in Fig. 2.

When adjusting for risk factors using the Framingham Risk Score (FRS) [21] and for sex, UMI remained associated with MACE (HR 2.55; 95 % CI 1.20-5.42; *p* = 0.015) whereas the association between RMI and MACE was weakened (HR 3.28; 95 % CI1.16-9.22; *p* = 0.025). HRs for FRS, and male sex were; 1.18; 95 % CI 1.05-1.32; *p* = 0.007; and 2.43; 95 % CI 1.13-5.22; *p* = 0.023 respectively.

C-statistics for Framingham risk score was 0.68; CI 0.58-0.77. Adding information on UMIs increased C-statistics to 0.75; CI 0.66-0.84 significantly (*p* = 0.0359). Adding information on UMIs to the Framingham risk score increased both IDI (0.068 (SE 0.022), *p* = 0.0020) and category-free NRI (0.67(SE 0.20), *p* = 0.0007) significantly.

The distribution of the events comprising MACE in the three groups is displayed in Table 2.

**Table 2:** Distribution of events regarded as major adverse cardiac events (MACE) in individuals with or without myocardial scars by CMR. (One individual could have several events)

	No MI (*n* = 182)	No MI vs UMI	UMI (*n* = 55)	UMI vs RMI	RMI (*n* = 11)	RMI vs No MI
Cardiac Death	2 (1 %)	ns	1 (2 %)	*p* = 0.001	3 (27 %)	*p* < 0.001
Non-fatal MI	15 (8 %)	ns	4 (7 %)	ns	3 (27 %)	ns
Revascularization	13 (7 %)	ns	6 (11 %)	ns	2 (18 %)	ns
Aquired Angina	10 (5 %)	ns	6 (11 %)	ns	3 (27 %)	*p* = 0.005

*No MI* no MI scar, *RMI* recognized myocardial infarction, i.e., MI scar in combination with MI diagnosis in medical records, *UMI* unrecognized myocardial infarction

Manifestations of other atherosclerotic disease (i.e. a diagnosis of carotid or renal artery stenosis or peripheral arterial disease) after the CMR examination were more frequently diagnosed in individuals with UMI (16 %, i.e. *n* = 9/55) than in individuals without MI scars (5 %, i.e. *n* = 10/182) (*p* = 0.009). (Table 3) After the CMR examination, individuals with RMI acquired other cardiac diagnoses (arrhythmias, congestive heart failure, or valvular disorders) more frequently than those without MI scars (*p* = 0.003). (Table 3)

**Table 3:** Frequency of cardiovascular symptoms and diagnoses (not regarded as major adverse cardiac events) acquired after 70 years of age in individuals with or without myocardial scars by CMR

	No MI (*n* = 182)	No MI vs UMI	UMI (*n* = 55)	UMI vs RMI	RMI (*n* = 11)	RMI vs No MI
Ischemic stroke	4 (2 %)	ns	3 (5 %)	ns	0	ns
Chest pain symptoms	40 (22 %)	ns	15 (27 %)	ns	5 (45 %)	ns
Other atherosclerosis	10 (5 %)	*p* = 0.009	9 (16 %)	ns	1 (5 %)	ns
Other cardiac morbidity	32 (18 %)	ns	15 (27 %)	ns	6 (54 %)	*p* = 0.003

*No MI* no MI scar, *RMI* recognized myocardial infarction, i.e., MI scar in combination with MI diagnosis in medical records, *UMI* unrecognized myocardial infarction

There were no differences in the frequency of hypertension, diabetes, ischemic stroke, or chest pain symptoms occurring after the CMR examination between the NoMI, UMI, and RMI groups. Lipid lowering medication was used equally in the three groups. There were no differences in medication between the individuals with UMI and those without MI scars.

## Discussion

In the present cohort the presence of a UMI entailed a more than double risk for MACE, also when adjusted for cardiovascular risk factors and sex. This observation confirms the results of other studies in selected [9–11, 13] and unselected populations [5]. Those studies were performed mainly in populations with important risk factors such as manifest atherosclerotic disease or diabetes, whereas the present study population was representative of the general population. Thus, the results of the present study imply that the prognostic impact of a UMI might be important regardless of other risk factors.

MACE was more prevalent in men (*n* = 25) than in women (*n* = 9) consistent with the known differences in clinically recognized cardiac morbidity and mortality [22]. However, MIs are more likely to be unrecognized in women than in men [22], partly because women more often present with atypical symptoms. [23–25] Consequently, there is a possibility that other major cardiac events may also be unrecognized in women. However, the groups are too small to allow any such conclusion from the present observations.

The weakened association between RMI and MACE that was seen when adjusting for risk factors and sex may be explained by the fact that 82 % (*n* = 9/11) of the individuals with RMI were men. The observed increased prevalence of cardiac death and angina in participants with RMI was expected, as RMI is a well known risk factor for cardiac events [26, 27]. Since other cardiac morbidity is associated with MI, it is not surprising that this too would be frequent in the RMI group.

Other studies have demonstrated that LGE-CMR improves risk stratification in patients [12] as well as in population based samples [5]. The results of the present study confirm this and may, thus, contribute in establishing CMR detected UMIs as a risk factor for MACE.

The risk for MACE may also be influenced by the size of the MI scar, since. UMIs have been observed to be generally smaller than RMIs [2, 13]. Thus, the presence of a smaller MI scar (i.e. a UMI) entails an increased risk for MACE compared to no MI scar, whereas the presence of a larger MI scar (i.e. an RMI) entails an even larger risk. These observations support the notion that a CMR detected UMI appears to represent an intermediate phenotype in the evolution of coronary heart disease [5].

In the ICELAND MI study participants with UMI were less likely to be treated with cardiovascular medications, such as statins than those with RMI [5]. No such difference could be detected in the present study, which may be due to low power since there were rather few participants with RMI. In the ICELAND MI study 36 % of the cohort had diabetes and participants with UMI were more frequently treated with cardiovascular medications than participants without MI scars [5]. In the present study cohort only 12 % had diabetes [2] and there were no differences in medication between the individuals with UMI and those without MI scars. Thus, the present cohort was slightly healthier (and/or not as well treated). Despite these differences HR for MACE in individuals with UMI was 2.55 in the present cohort and 1.45 in the ICELAND MI study [5], implying that UMI may be an important risk factor also in otherwise healthy individuals. Consequently, individuals with a CMR detected UMI might benefit from cardioprotective medication regardless of other cardiovascular risk factors. These individuals might need surveillance and preventive measures.

At baseline the prevalence of atherosclerotic disease was increased in participants with RMI, but not in participants with UMI compared to those without MI scars in the present sample [28]. However, during the follow-up period atherosclerotic diseases were more frequently diagnosed in participants with UMI, but not in participants with RMI compared to those without MI scars. (Table 3) The observation that individuals with RMI frequently had other atherosclerotic diseases already at baseline was expected. Furthermore, individuals with these diagnoses are most likely under surveillance and treatment which might explain why new atherosclerotic manifestations were not frequently detected during follow-up.

The observation that participants with UMI had an increased prevalence of atherosclerotic disease at follow-up but not at baseline suggests a more slowly progressing disease in these individuals, compared to in those with RMI. The fact that MACE was twice as prevalent in individuals with UMI and more than four times as prevalent in individuals with RMI compared to those without MI scars in the present cohort (Fig. 1) may reflect an evolvement from subclinical to manifest atherosclerosis.

The present study was limited by the fact that only elderly Caucasians were studied and consequently the observations may not be applicable to other ethnic or age groups. The participants were consecutively invited from a randomized cohort of community-living individuals for the first CMR, and those who participated were invited for a second CMR five years later. Creating a subsample of a subsample, which is unavoidable in a follow up study, might introduce a selection bias. However, the cardiac morbidity of the PIVUS study participants did not differ significantly from that of the background population [15], and basic characteristics and major cardiovascular risk factors did not differ between the present cohort and the entire PIVUS population [15], except that there were fewer current smokers among the individuals of the present study. Thus, the present cohort might be slightly healthier than the background population but it is unlikely that this has affected the results in any important way. Another limitation was that the individuals with RMI were rather few (*n* = 11). However, the main purpose of this study was to investigate the prognostic impact of UMI.

## Conclusions

The presence of a CMR detected UMI entailed a more than double risk for MACE in community living 70-year-old individuals, also when adjusting for cardiovascular risk factors and sex.

## Abbreviations

CMR, cardiovascular magnetic reosnance; ECG, electrocardiography; FRS, framingham risk Score; MACE, major adverse cardiac events; RMI, recognized myocardial infarction; UMI, unrecognized myocardial infarction
